# Assessing Plasmin Generation in Health and Disease

**DOI:** 10.3390/ijms22052758

**Published:** 2021-03-09

**Authors:** Adam Miszta, Dana Huskens, Demy Donkervoort, Molly J. M. Roberts, Alisa S. Wolberg, Bas de Laat

**Affiliations:** 1 Synapse Research Institute, 6217 KD Maastricht, The Netherlands; d.huskens@thrombin.com (D.H.); 1846280donkervoort@zuyd.nl (D.D.); 1819119roberts@zuyd.nl (M.J.M.R.); b.delaat@thrombin.com (B.d.L.); 2 Department of Pathology and Laboratory Medicine and UNC Blood Research Center, University of North Carolina at Chapel Hill, Chapel Hill, NC 27599, USA; alisa_wolberg@med.unc.edu

**Keywords:** plasmin, plasmin generation, fibrinolysis

## Abstract

Fibrinolysis is an important process in hemostasis responsible for dissolving the clot during wound healing. Plasmin is a central enzyme in this process via its capacity to cleave fibrin. The kinetics of plasmin generation (PG) and inhibition during fibrinolysis have been poorly understood until the recent development of assays to quantify these metrics. The assessment of plasmin kinetics allows for the identification of fibrinolytic dysfunction and better understanding of the relationships between abnormal fibrin dissolution and disease pathogenesis. Additionally, direct measurement of the inhibition of PG by antifibrinolytic medications, such as tranexamic acid, can be a useful tool to assess the risks and effectiveness of antifibrinolytic therapy in hemorrhagic diseases. This review provides an overview of available PG assays to directly measure the kinetics of plasmin formation and inhibition in human and mouse plasmas and focuses on their applications in defining the role of plasmin in diseases, including angioedema, hemophilia, rare bleeding disorders, COVID-19, or diet-induced obesity. Moreover, this review introduces the PG assay as a promising clinical and research method to monitor antifibrinolytic medications and screen for genetic or acquired fibrinolytic disorders.

## 1. Introduction

Hemostasis is a process of regulated balance between clot formation and clot lysis, aimed at preventing blood loss while maintaining vascular patency. Activation of coagulation factors and platelets leads to thrombin generation (TG) and ultimately, clot formation. Subsequently, the fibrinolytic system is crucial for clot remodeling and lysis. Perturbation of these processes can lead to bleeding disorders or thrombotic events. Although several tools to assess fibrinolysis have been developed, few methods are available to quantify the amount of plasmin generated. The scope of this review is to outline methods to measure plasmin generation (PG) and highlight applications of these assays in human samples and mouse models of disease.

## 2. Plasmin and the Molecular Mechanism of Plasmin Generation

**Figure 1:**
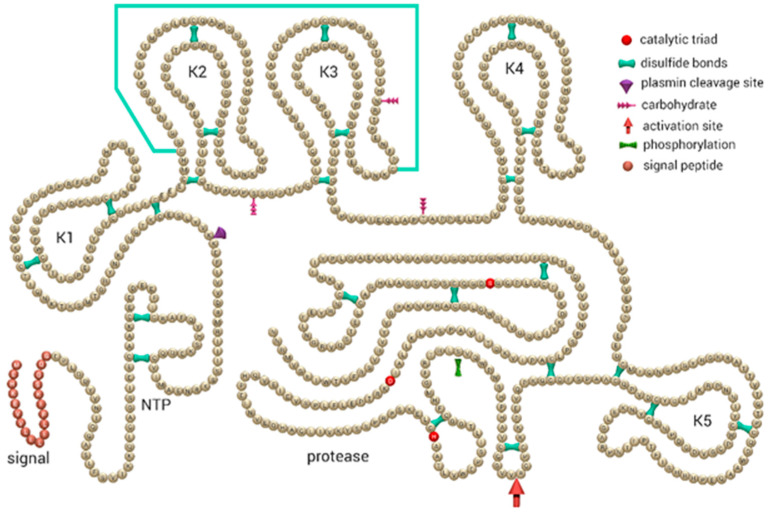
Schematic representation of the primary structure of human glutamic (Glu)-plasminogen. The catalytic triad (His603, Asp646, and Ser741) within the protease domain, the activation site (Arg561–Val562), and the 24 disulfide bridges as well as the signal peptide are indicated. NTP, N-terminal peptide; K1–K5, kringles 1–5. Previously published [3] and adapted from Schaller and Gerber [4].

Plasmin is a major fibrinolytic protease generated by converting its zymogen plasminogen by fibrinolytic activators in the presence or absence of fibrin. Plasminogen is synthesized primarily in the liver and circulates in plasma at a concentration of approximately 1.5 μmol/L [1]. Glu-plasminogen is a single chain glycoprotein with glutamic acid (Glu) as the NH_2_ residue, consisting of an N-terminal activation peptide (NTP), five kringle domains (K1–K5), and a serine protease domain containing the catalytic triad (Figure 1) [1,2].

Lys-plasminogen is a truncated plasmin-derived form that lacks the NH_2_-terminal 1–77 peptide and is produced during fibrinolysis [5]. Whereas Glu-plasminogen assumes a closed conformation because of intramolecular links between the NH_2_-terminal peptide and kringle 5 domain, Lys-plasminogen has a more open conformation. The conformational change of Glu-plasminogen to Lys-plasminogen during fibrinolysis not only renders Lys-plasminogen more readily activated by plasminogen activators, but also endows Lys-plasminogen with a higher affinity for fibrin than its intact precursor. Of the five kringle domains in plasminogen, kringles 1, 4, and 5 are reported to be the major mediators of the plasminogen/fibrin interaction [6,7].

**Figure 2:**
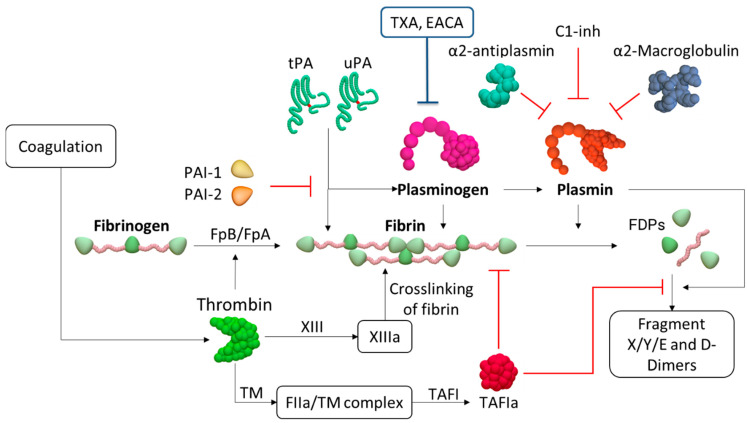
A schematic representation of the fibrinolytic system. Fibrinolysis is initiated when the product of coagulation, thrombin, cleaves fibrinopeptides A and B from fibrinogen, leading to fibrin network formation. Thrombin activates factor XIII that crosslinks fibrin. Tissue plasminogen activator (tPA) or urokinase-type plasminogen activator (uPA) converts plasminogen into plasmin. Plasmin cleaves fibrin into fibrin degradation products (FDPs), including D-dimer. Direct plasmin inhibitors are α_2_-antiplasmin, α_2_-macroglobulin, and C-1 esterase inhibitor (C1-INH). Plasmin formation is also downregulated by several inhibitors, including plasminogen activator inhibitor 1 (PAI-1) which inhibits tPA and uPA. Interaction of thrombin with thrombomodulin (TM) activates thrombin-activated fibrinolysis inhibitor (TAFI). Activated TAFI (TAFIa) reduces plasminogen activation by cleaving C-lysines from fibrin and FDPs. Activation of plasmin can also be inhibited by lysine analogues tranexamic acid (TXA) and ε-aminocaproic acid (EACA).

Tissue plasminogen activator (tPA) and urokinase-type plasminogen activator (uPA or urokinase) are plasminogen activators. TPA is secreted predominantly from endothelial cells triggered by local stimuli, including thrombin activity, bradykinin (a product of high molecular weight kininogen cleavage by kallikrein), and shear stress. TPA-mediated PG is initiated when thrombin, the central enzyme in coagulation, cleaves fibrinopeptides A and B from fibrinogen and enables fibrin network formation (Figure 2). This process exposes cryptic domains in fibrin(ogen) that allows for binding of both plasminogen and a plasminogen activator tPA to fibrin via C-Lysine binding sites. Exposure of these sites is driven primarily by the interaction between complementary sites of the D and E regions [1]. The catalytic efficiency of plasminogen activation by tPA is 3 orders of magnitude greater in the presence of fibrin than it is in its absence and 2 orders of magnitude greater in the presence of fibrinogen. uPA is expressed by endothelial cells, macrophages, and renal epithelial cells and activates plasminogen independent of fibrin, when it interacts with the cellular receptor urokinase protease-activated receptor (uPAR) [8,9]. uPA catalytic efficiency for plasminogen activation is similar in the presence and absence of fibrin or fibrinogen.

Previous studies localized a set of specific low-affinity tPA- and plasminogen-binding sites in each D region of fibrin(ogen). The tPA-binding site includes residues γ312-324 and the plasminogen-binding site includes residues Aα148-160. High-affinity tPA- and plasminogen-binding sites were identified in the compact portion of each fibrin(ogen) αC-domain within residues Aα392-610 [10]. Plasminogen activation by fibrin-bound tPA or uPA bound to uPAR occurs via cleavage of the Arg561-Val562 of both proenzymes, which leads to the formation of the two-chain enzyme plasmin composed of an N-terminal heavy chain (12–65 kDa) and a C-terminal light chain (25 kDa) [5]. Subsequently, plasmin cleaves fibrin, producing fibrin degradation products (FDPs), including D-dimer.

Several mechanisms downregulate the fibrinolytic response. Three major direct plasmin inhibitors are α_2_-antiplasmin, α_2_-macroglobulin, and C-1 esterase inhibitor (C1-INH) [11]. The most effective inhibitor, α_2_-antiplasmin, circulates in plasma at a high concentration and is also a constituent of platelet α-granules [12,13]. Hence, plasmin in blood or the vicinity of a platelet-rich thrombus is rapidly neutralized by α_2_-antiplasmin [1]. α_2_-macroglobulin, synthesized by endothelial cells and macrophages and present in platelet α-granules, inhibits plasmin with approximately 10% of the efficiency of α_2_-antiplasmin [1]. C1-INH, the key regulator of the complement and contact systems, is also involved in the control of blood coagulation and fibrinolysis. C1-INH may inhibit both plasmin [14] and tPA [15]. C1-INH is thought to inhibit plasmin according to the general mechanism of serpin action; however, little is known about the Cl-INH/plasmin interaction. It has been shown that plasmin can cleave C1-INH in multiple sites leading to the formation of two derivatives of C1-INH migrating at approximately 96 kDa and 83 kDa [11,16,17,18]. Plasmin formation is also regulated by several inhibitors of plasminogen activation, including plasminogen activator inhibitor -1 (PAI-1) and plasminogen activator inhibitor -1 (PAI-2) which inhibit tPA and uPA. PAI-1 is the most important and most rapidly acting physiological inhibitor of tPA and uPA. Significant levels of PAI-2 are found in human plasma only during pregnancy. Thrombin-activated fibrinolysis inhibitor (TAFI) is expressed in the liver and is present in platelets [19]. Its activation by thrombin is accelerated ~1250-fold in the presence of thrombomodulin [1]. Activated TAFI reduces plasminogen activation by cleaving C-lysines from fibrin and FDPs. Because these residues are binding sites for plasminogen and tPA, active TAFI potently attenuates fibrinolysis. A schematic representation of fibrinolysis is shown in Figure 2.

## 3. Diseases Associated with Plasmin Generation

PG can be altered by several processes, such as impaired fibrin formation, altered levels of fibrinolytic factors (for instance PAI-1, α_2_-antiplasmin, fibrinogen, TAFI, tPA, or uPA), or the interaction of plasmin(ogen) with cellular receptors [20,21,22,23] or pathogens during infections [24]. These processes play a key role in fibrinolysis and hemostasis, where plasmin targets fibrin, fibrinogen, factors V(a), VIII(a), and X [25,26], complement component 3 (C3), complement component 5 (C5) [27], and vitronectin [28]. Plasminogen and plasmin also regulate other physiological and pathological events (Table 1). The roles of plasminogen and plasmin in diseases and therapeutic treatments have been previously reviewed [29,30,31,32,33,34,35].

## 4. Fibrinolytic Assays Are Overshadowed by Clotting Tests

Methods to assess blood coagulation dominate the clinical laboratory diagnostic arsenal. Coagulation assays, such as the prothrombin time and activated partial thromboplastin time, are widely available and standardized (e.g., the international normalized ratio, derived from the prothrombin time) for routine use [63]. Although not yet routinely available for diagnostics, major developments in the field of global coagulation assays have taken place over the past decades, in particular in the development of TG tests [64,65,66].

Equivalent systems for measuring fibrinolysis have lagged. One reason may be that diseases with a higher prevalence naturally drive research and development into tests that aid in the diagnosis and/or monitoring of disease progression. Deficiencies or dysfunction of components of the fibrinolytic system appear to be less prevalent compared to disturbances in the amount or function of coagulation factors (e.g., factor VIII and factor IX in hemophilia A and B, respectively), von Willebrand Factor, and platelets [67]. However, one could argue that the relative dearth of routine standardized fibrinolytic assays may have led to an underestimation of the prevalence of disturbances in fibrinolysis, for instance in individuals with unexplained bleeding [68]. Enhanced methods to define fibrinolytic function may fill this gap and illuminate previously unrecognized deficiencies that lead to clinically relevant disorders.

## 5. Assays That Measure Fibrinolytic System Components in Blood

Several assays are available to assess concentrations of proteins within the fibrinolytic pathway [69]. Most methods measure either the concentrations of circulating fibrinolytic or antifibrinolytic proteins (e.g., tPA, plasminogen, α_2_-antiplasmin, PAI-1, TAFI, and C-1-INH esterase inhibitor) or the products of fibrinolysis (e.g., plasmin-α_2_-antiplasmin (PAP) complexes and D-dimer). These measurements can be used to infer recent fibrinolytic events in vivo and have successfully uncovered enhanced fibrinolytic activity in multiple diseases such as obesity, liver cirrhosis, and thrombotic thrombocytopenic purpura [44,45,70,71]. However, while these biomarkers can reveal endogenous fibrinolytic events, they do not provide information on the fibrinolytic potential of the blood.

## 6. Functional Assays That Measure Fibrinolytic Potential

Measuring viscoelastic or turbidimetric properties of fibrin during its formation and lysis provides information on global fibrinolytic potential [72,73].

Thromboelastography is the method that focuses on the viscoelastic changes of whole blood in the presence of coagulation factors. This method uses special equipment, consisting of a recording device, a cup for whole blood, and a pin. Two techniques that use this approach are the thromboelastograph (TEG) and rotation thromboelastometer (ROTEM) [74,75,76]. In ROTEM, the pin oscillates within the cup, whereas in TEG, the cup is rotated around the fixed pin. Both methods provide information on coagulation (e.g., clotting time, clot formation time, time to maximum amplitude (TMA), or maximum clot firmness (MA)), as well as fibrinolysis (e.g., maximum lysis (MA) or lysis at 30 min (LY30)). Although coagulation and fibrinolysis can be monitored simultaneously, the major disadvantages of this method are high cost of the equipment and poor reproducibility [77].

Another method that measures viscoelastic properties of whole blood is the ReoRox [78,79]. In this assay, the free oscillation and movement of the cup with whole blood are recorded by an optical detector. The changes of damping and frequency of the oscillation correlate with viscosity and elasticity, respectively, which are presented as viscosity and elasticity curves.

Rheometry allows for measurement of viscosity changes during fibrin formation and lysis while applying a linear shear flow. The recent modification of this method to introduce a fluorometer into a “cone and base principle”-based instrument allows the simultaneous measurement of TG and fibrinolysis [72]. Interestingly, in this method, the coagulation and fibrinolysis of both plasma and whole blood can be measured under a continuous flow.

Turbidity is a standard assay to monitor fibrin formation and lysis by recording changes in optical density using spectrophotometry [73]. This method detects the absorbance of plasma triggered with tissue factor (TF) or by thrombin. Turbidity can be performed in the presence of plasminogen activators (tPA or uPA). The most common method that uses turbidity is the overall hemostasis potential (OHP) assay. In this assay, the plasma is mixed with TF, phospholipids, and CaCl_2_, and the absorbance is measured as a function of time at 405 nm in a 96-well microplate. From the curve, multiple parameters describing fibrin formation and fibrinolysis can be obtained (e.g., onset (time that is required for clot formation), maximum absorbance at plateau (maximum fibrin formation), time to plateau (time to maximum absorbance at plateau), maximum rate of turbidity increase, and clot lysis time (CLT, most frequently defined as the time from 50% of maximum clotting to 50% lysis)).

Viscoelastometric and turbidimetric assays demonstrate an advantage over PG assays since they measure fibrinolytic activity in whole blood. However, their global nature makes it difficult to define the origin of fibrinolytic abnormalities (i.e., abnormal fibrin formation or structure vs. abnormal generation or function of the fibrinolytic system). Other problems include the storage of whole blood, assay standardization, and in the case of turbidimetric assays, the absorbance of light by hemoglobin [77].

## 7. Assays That Measure Plasmin Generation Kinetics

Several groups have developed assays to determine plasmin capacity in plasma by initiating coagulation and fibrinolysis with tissue TF, tPA, phospholipids, and CaCl_2_. The Nijmegen hemostasis assay (NHA) measures both TG and PG in a single well using two fluorescent substrates (Bz-beta-Ala-Gly-Arg-7-amino-4-methylcoumarin (AMC) and bis-(CBZ-L-phenylalanyl-L-arginine amide)-rhodamine for thrombin and plasmin, respectively) with non-interfering fluorescent excitation and emission spectra (thrombin: Excitation λ 355 nm; emission λ 460 nm; plasmin-excitation λ 485 nm; emission λ 520 nm) [80]. This method is performed in platelet-poor plasma (PPP) triggered with phospholipids, TF (0.3 pM), and tPA (0.38 μg/mL). Thrombin and plasmin proteolytic activities are calculated by comparing the arbitrary fluorescence values to a calibration curve prepared with known amounts of human α-thrombin and human plasmin, respectively [80,81,82,83]. To describe the proteolytic activity of plasmin, three parameters are defined by calculating the first derivative of the fluorescence signal: The plasmin peak-height (nM), the fibrin lysis time (FLT, minutes), and plasmin potential (nM*minute).

The simultaneous thrombin and plasmin generation (STA) assay measures thrombin and PG in two separate wells using the AMC fluorometric substrates Boc-Val-Pro-Arg-AMC and Boc-Glu-Lys-Lys-AMC, respectively (excitation λ 360 nm; emission λ 460 nm) [83]. This method is performed in 96-well microplates and the final concentrations of tPA and TF in the reaction mixture are 0.45 ug/mL and 5 pM, respectively. In this assay, platelet-poor plasma is mixed with a solution containing TF and tPA. Raw fluorescence data obtained from this assay are determined by subtracting the fluorescence reading of the blank well (Tris-buffered saline instead of plasma). No calibrator is used, and results are presented as a function of fluorescence intensities rather than concentrations. Parameters obtained from this assay are: Lag time to PG, maximum amplitude of PG, maximum velocity of PG, time to the maximum velocity of PG, time to maximum amplitude of PG, and area under the first-derivative curve of PG. Matsumoto et al. modified this assay by choosing another thrombin substrate (Z-Gly-Gly-Arg-AMC) and by introducing the use of the standard curve prepared by using serial dilutions of α-thrombin and plasmin [84]; PG parameters calculated are lag time, time to peak, and endogenous potential.

Another assay that measures TG and PG in parallel was described by Tarandovsky et al. and uses Z-Gly-Gly-Arg-AMC and Boc-Glu-Lys-Lys-AMC substrates for thrombin and plasmin, respectively (excitation λ 360 nm; emission λ 460 nm) [85]. This assay, called the simultaneous thrombin plasmin generation assay (STPGA), is performed in platelet-poor plasma using TF, phospholipids, tPA, and CaCl_2_ as a trigger. The final concentration of TF is 4.5 pM and of tPA is 0.7 μg/mL. No calibrator is used but another method is employed to correct for the inner filter effect (adsorption of light by non-fluorescent proteins in plasma) and substrate consumption [86]. The plasmin concentration is calculated using the Michaelis–Menten equation using the obtained values of the AMC production rate. Parameters for PG are peak concentration and average plasmin production rate before reaching the peak [85]. In an interspecies comparison, this group showed that TG and PG parameters from baboon and rhesus macaque plasma approximate that of humans, while other species (pigs, rats, and rabbits) differed from human [87].

Each of these assays has revealed intriguing clinically-relevant information that is discussed below, but has potential limitations. Some assays use plasmin as a calibrator; however, they do not consider plasmin inhibition by endogenous plasmin inhibitors. The STA assay also does not correct for substrate consumption during the reaction process, or inner filter effects. Finally, the lack of substrate specificity (e.g., the NHA uses a plasmin substrate that can also be cleaved by thrombin and activated Factor X), makes it challenging to attribute signal to plasmin activity [80]. These issues limit the ability to quantify functional plasmin activity generated during the reaction.

Recently, a calibrated PG assay (PGA) to monitor plasmin formation and inhibition in plasma was developed [44,88]. This assay is based on the established approach employed by calibrated automated thrombography, and overcomes several limitations associated with the earlier methods. Firstly, by using the α_2_-Macroglobulin-plasmin complex as a calibrator, the PGA not only corrects for substrate exhaustion and the inner filter effect, but also eliminates inhibition of the calibrator by plasmin inhibitors. Secondly, this assay uses a plasmin-specific fluorogenic substrate Boc-Glu-Lys-Lys-AMC, eliminating problems associated with substrate specificity [89,90,91]. Curves obtained by this method provide quantitative information on the kinetics of plasmin formation and inhibition during fibrinolysis and are expressed in molar concentrations. The final concentration of TF in this assay is either 0.5 or 1 pM and either 0.31 or 1.25 μg/mL of tPA. Multiple parameters can be obtained from the PG kinetic curve, including lag time (time needed for plasmin concentration to reach 6 nM of the peak concentration), velocity (rate of plasmin formation), peak height, time to peak (ttpeak, time needed to reach maximum plasmin activity), and endogenous plasmin potential (EPP, the overall activity of plasmin formed during fibrinolysis). PGA can be performed both in human and mouse plasmas and is sensitive to concentrations of recombinant tPA, plasminogen, and α_2_-antiplasmin. Furthermore, this assay is also strongly dependent on fibrin polymerization, but not fibrin crosslinking (described below) [44,88]. The overview of the existing PG assays is shown in Table 2.

## 8. Plasmin Generation in Health and Disease

Although several PG assays have been developed, to date not many studies have investigated PG in diseases associated with abnormal fibrinolysis that lead to bleeding or thrombotic events. This section highlights major applications of PG assays in human samples and mouse models of disease. We first introduce published studies on PG as applied to bleeding and thrombotic diseases. We also describe a PG assay used to study a mouse model of diet-induced obesity and antifibrinolytic use in the post-partum period.

Bleeding from congenital deficiencies of fibrinolysis inhibitors, such as PAI-1 and α_2_-antiplasmin are rare [96,97]. Van Geffen et al. used the NHA assay to study patients with deficiencies of plasminogen and PAI-1, and distinct abnormalities in both PG and TG were found [92]. The same assay was used to study PG in hemophilia A patients before and after administration of a single dose of 25–50 IU/kg standard half-life factor VIII concentrate [93]. Patients with severe hemophilia A have higher plasmin production than patients with mild hemophilia or healthy controls, but hyperfibrinolysis observed in these patients is normalized after factor VIII supplementation. In contrast, using a different PG assay, Matsumoto et al. did not find a relationship between PG and factor VIII:C, noting that the peak and ttPeak were not significantly different between severe hemophilia A patients and plasma prepared from 20 healthy individuals [84].

Rare bleeding disorders arise from inherited deficiencies of certain coagulation factors, including fibrinogen, prothrombin, or factor (F)V, VII, X, XI, and XIII. A retrospective analysis of PG in patients with rare bleeding disorders was performed using the NHA by van Geffen and colleagues [81]. In this study, PG was measured in 41 patients affected with deficiencies in prothrombin, (F)V, VII, X, XIII, or fibrinogen. Their results showed that prothrombin deficiency results in delayed PG, normal FLT, and reduced plasmin peak and potential when compared to controls. In contrast, FX, FVII, and FXIII deficiency showed no difference in FLT, plasmin peak-height, or plasmin potential. Afibrinogenemic patients demonstrated reduced NHA plasmin peak-height, and no detectable FLT. Statistical analysis showed significant differences between bleeding tendencies based on plasmin potential. Plasmin potential was lower in patients with major bleeding compared with those that had only minor bleeding.

Impaired fibrinolysis may also lead to venous and/or arterial thrombosis [98]. Most reports of associations between abnormal fibrinolysis and thrombotic risk have assessed levels and/or activity of individual fibrinolytic proteins, such as PAI-1, tPA [99,100], α_2_-antiplasmin [101], and TAFI [102]. Levels of the latter two proteins are associated with a mildly increased risk of development of arterial and venous thrombosis, respectively [102]. Complexes of these proteins (e.g., tPA/PAI-1 complex, PAP complex [103]), as well as D-dimer formation and depletion of circulating plasminogen and fibrinogen, can be used as markers of ongoing fibrinolysis. To date, few studies have investigated PG in prothrombotic settings. Bouck et al. showed that patients with either coronavirus disease-2019 (COVID-19) or sepsis have elevated fibrinogen, D-dimer, soluble thrombomodulin, and PAP complexes, but detected enhanced PG in patients with COVID-19, and delayed PG in plasma from patients with sepsis [94]. In contrast, de Jongh et al. did not detect higher PG in COVID-19 patients [95]. Differences in these studies may reflect the relatively small sample size of each, or other aspects of the patient populations.

Heterogeneity of human blood makes it difficult to attribute changes in fibrinolytic components to bleeding or thrombosis. However, similarities in fibrinolytic system components in humans and mice enable studies of this system in defined in vivo settings [104,105,106]. Moreover, the ability to perform genetic manipulations in mouse models enables targeted experiments to define specific contributions of fibrinolytic proteins in health and disease. For example, experiments to cross fibrinogen deficient mice (*Fbg^−/−^*) with plasminogen deficient mice (*Plg^−/−^*) showed that loss of fibrinogen rescues many of the abnormalities associated with plasminogen deficiency, suggesting that abnormalities in *Plg^−/−^* mice stem primarily from excessive fibrin deposition [107].

One of the most prevalent prothrombotic risk factors associated with altered expression of coagulation factors and decreased fibrinolysis is obesity. Expression of the endogenous tPA inhibitor PAI-1 strongly correlates with body mass index, and PAI-1 has an important role in venous thrombosis and resistance of platelet-rich arterial thrombi to lysis [108,109]. Although several studies have identified mechanisms that enhance TG and fibrin formation in obesity, less is known about dynamics of plasmin. Miszta et al. applied PGA to an experimental setting of diet-induced obesity in mice fed a control diet (CD) or high-fat diet (HFD) and detected significantly delayed PG in plasma in HFD-fed mice [44]. Although PG parameters significantly correlated with both total and active PAI-1, C1-INH, and TAFI, changes observed by PG were not explained by elevated levels of these proteins. Additionally, proteins that have a strong effect on PG, such as plasminogen, α_2_-antiplasmin, and fibrinogen, were not elevated in plasma from HFD-fed mice. Interestingly, this study revealed a thrombomodulin- and TAFI-dependent mechanism that delays PG in plasma from HFD-fed mice. Identification of this mechanism uncovers new pathologic pathways relating HFD and obesity with enhanced fibrin stability in a prothrombotic setting.

The relationship between PG and fibrin formation was extensively investigated with PGA using genetically modified mice by Miszta at al. [44]. These results showed that PG was not detected in plasma from *Pl^−/−^* mice, confirming substrate specificity for plasmin. The relationships between fibrin formation and PG were characterized using plasmas from wild-type mice and mice with deficiencies or abnormalities in fibrinogen concentration or fibrin assembly. These experiments showed that compared to *Fga^+/+^* mice, partial deficiency in *Fga^+/^^−^* resulted in significantly decreased fibrin formation and reduced the PG velocity, peak, and EPP. As expected, *Fga^−/−^* mice did not form fibrin or generate plasmin. Plasmas from mice expressing normal levels of a mutant fibrinogen that cannot polymerize (*Fgn^AEK^*) showed decreased and no fibrin formation in plasma from *Fgn^WT/AEK^* and *Fgn^AEK/AEK^* mice, respectively, indicating the dependence of this reaction on fibrin. Moreover, compared to *Fgn^WT/WT^* mice, plasma from *Fgn^WT/AEK^* mice possess reduced peak and EPP, and plasma from *Fgn^AEK/AEK^* mice did not support PG. Interestingly, no difference in fibrin formation or PG in plasmas from *F13a1^+/+^*, *F13a1^+/−^*, and *F13a^−/−^* mice was observed, confirming previous findings performed in plasma from human patients with rare bleeding disorders using NHA assay [81]. These findings showed that the PG assay is strongly dependent on fibrin polymerization, but not fibrin crosslinking.

PG assays may also be useful tools to monitor treatment with anti-fibrinolytic agents. Tranexamic acid (TXA), a lysine analogue, inhibits binding of both zymogen plasminogen and its active form plasmin to fibrin [110,111]. Effects of TXA are typically studied using turbidity, ROTEM, or TEG [112]. These assays provide combined information on fibrin formation and fibrinolysis; however, they do not differentiate between TXA’s ability to reduce plasmin cleavage of fibrin from its ability to block tPA-mediated generation of plasmin. Miszta et al. used the PGA to characterize the effects of TXA administered in vitro and in vivo [88]. The results revealed exquisite sensitivity of the PGA to pharmacologically relevant concentrations of TXA added to plasma in vitro, as well as in plasmas from women administered TXA during cesarean delivery. Notably, effects of TXA on PG parameters measured in plasma were similar to parameters obtained from ROTEM performed in whole blood; however, the PGA had increased sensitivity to low (<10 µg/mL) TXA. Other PG parameters (time-to-peak, velocity, and peak) showed better correlation with TXA concentration and less variability compared to either ROTEM LI30 or maximum lysis [88]. Since TXA is used as an antifibrinolytic in a number of clinical situations (e.g., shock [113], trauma [114], cardiopulmonary bypass [115], postpartum hemorrhage [116], and malignancy [59,117,118,119]), the PGA may have broad utility for identifying fibrinolytic dysfunction and optimizing antifibrinolytic therapy.

## 9. Summary and Future Directions

Although multiple PG assays have been developed to assess abnormal fibrinolysis in bleeding or thrombotic diseases, the (patho)physiological effects of plasmin in many diseases is still unclear. The development of functional methods to quantify PG may fill knowledge gaps necessary to understand the relationships between abnormal fibrin dissolution and disease pathogenesis. PG tests can be used to evaluate the specific characteristics of fibrinolysis, such as levels of fibrinogen, plasminogen, plasminogen activators and inhibitors, the capacity of fibrin to generate plasmin or to provide information about the initiation, amplification/propagation, and resolution phases. Although in vivo PG measured by D-dimer or PAP complexes represents the pathological activation of fibrinolytic system, the ex vivo PGA aims to evaluate the endogenous capacity of fibrinolysis to predict hemorrhagic or thrombotic risk. To date, PG assays have been designed to measure plasmin capacity in plasma and not whole blood. Therefore, the adjustment of these assays to include cells will be extremely important. Cells play a significant role in the regulation of fibrinolysis via secretion and synthesis of plasminogen activators and inhibitors. For example, monocytes release urokinase that activates plasminogen in the presence of uPAR and the transmembrane plasminogen receptor (PlgR-KT) [120,121], and this results in more rapid activation and enhanced thrombolysis [122,123,124]. Platelet-derived plasminogen is retained on the activated platelet membrane and drives local fibrinolysis by enhancing cell surface-mediated plasminogen activation [125]. Furthermore, PAI-1 is released from monocytes and platelets [126]. On leukocytes, integrin α_M_β_2_ binds both plasminogen and urokinase, serving as a cofactor for PG [127]. Other plasminogen cellular receptors include α-enolase [20,21,22], actin [128], αIIbβ3 [129], α5β1 [130], cytokeratin 8 [131], S100A10 [23], TIP49a [132], and histone H2B [133], as well as amphoterin [134] and GP330 [135]. Previous studies also demonstrated a strong interaction of plasminogen with the cell surface of pathogens during infection [24] and the role of plasmin in activation of matrix metalloproteases MMP-1 and MMP-9 [136].

Another potential application of PG assays is to investigate the role of microvesicles in fibrinolysis. All eukaryotic and prokaryotic cells as well as cancer cells release microvesicles. Microvesicles not only represent procoagulant activity but can also influence the fibrinolytic system [137]. Endothelial microvesicles contain tPA, while leukocyte microvesicles express urokinase and uPAR. Interestingly, plasminogen activators are not found on erythrocytes or platelet-microvesicles [138]. Conversely, platelet-microvesicles release PAI-1 [139,140]. In addition to expressing activators and inhibitors of PG, microvesicles also provide a surface for plasmin. Plasmin bound to cell surfaces is protected from inhibition by α_2_-antiplasmin [140]. Future studies and development of PG assays should focus on the role of cells in PG and the contribution to pathological conditions.

Antifibrinolytic therapy is used in many settings including trauma, postpartum hemorrhage, cardiac surgery, and surgery, and has been previously reviewed [141]. A major concern of the use of antifibrinolytic therapy is its potential role in the development of thrombotic events [142,143]. Application of PG assays might be a promising tool to define potential prothrombotic and antifibrinolytic mechanisms in these clinical scenarios and fill this gap.

Based on the already published studies, PG assays have promising clinical and research potential, in monitoring antifibrinolytic medications, screening for genetic or acquired fibrinolytic disorders, or evaluating changes in fibrinolytic system due to abnormalities in the levels of fibrinolytic factors, both in human and mouse plasmas [44,81,85,88,92,93,94,95,144]. Measurement of these changes may better reflect the hemostatic phenotype compared to routine tests (such as D-dimer, PAI-1, PAP complexes and tPA levels, or turbidity, ROTEM and TEG). In this sense, for patients with thrombosis or bleeding in which conventional screening tests are normal, the presence of abnormal PG should be considered. Although PG assays are not yet available for clinical use due to lack of standardization and validation, work is ongoing to advance the development of these assays for both laboratory and experimental studies.

## Figures and Tables

**Table 1:** Overview of the processes and diseases together with key findings where plasminogen and plasmin are involved.

Processes and Diseases	Key Finding(s)	Reference
Wound healing	Plasminogen is required for the repair of skin wounds in mice; plasmin-mediated proteolysis plays a central role in cardiac wound healing after myocardial infarction in mice	[36,37]
Oncogenesis and metastasis	S100A10 is a key regulator of cellular plasmin production; plasminogen-binding proteins were detected in the plasma membranes of the human breast cancer cell line MDA-MB-231	[23,38,39]
Degradation of extracellular matrix, muscle regeneration	Inhibition of plasmin activity with α_2_-antiplasmin results in decreased myoblast fusion and differentiation in vitro	[40]
Cell migration or tissue remodeling	α-enolase constitutes a receptor for plasminogen on several leukocyte cell types, serving to localize and promote plasminogen activation	[20,21]
Apoptosis	Disruption of neuron–ECM interaction via tPA/plasmin degradation of laminin sensitizes hippocampal neurons to cell death	[41,42]
Liver diseases, mainly cirrhosis	Changes in tPA, PAI-1, and active PAI-1, leading to hyperfibrinolysis	[43]
Obesity	Thrombomodulin-dependent activation of TAFI results in delayed PG	[44]
Thrombotic thrombocytopenic purpura	Plasmin levels are increased during acute TTP, although limited via suppression by α_2_-antiplasmin and PAI-1	[45]
Von Willebrand factor disease	Plasmin is able to proteolyze von Willebrand factor	[3]
Stroke	Plasminogen has a protective effect on the ischemic brain by improving the clearance of macrovascular thrombi and restoring reperfusion	[46]
Cardiovascular diseases	Plasmin-α_2_-antiplasmin complex levels predict acute myocardial infarction in the elderly	[47]
Trauma	Changes after severe injury lead to trauma-induced coagulopathy and coagulation changes that cause hyperfibrinolysis	[48]
Antiphospholipid syndrome	Elevated levels of PAI-1; presence of antibodies inhibiting plasmin and tPA	[49,50]
Gum disease	Miropin expressed by Tannerella forsythia is an efficient inhibitor of plasmin	[24]
Alzheimer disease	Plasmin contributes to the catabolism and clearance of neurotoxic β-amyloid (Aβ)	[51]
Central nervous system	tPA is a modulator of neurotransmission and the synaptic plasticity process; tPA/Plasminogen axis contributes to excitotoxic neuronal degeneration	[52,53,54]
Coronavirus disease-2019	Enhanced plasmin generation potential in plasma	[55]
Hereditary angioedema	Plasmin cleaves and activates factor XII associated with hereditary angioedema	[56]
Cardiopulmonary bypass	Increased D-dimer and PG, measured by plasmin-α_2_-antiplasmin complexes and antiplasmin level	[57]
Placenta disorders	PAI-1 is responsible for inhibiting extracellular matrix degradation, thereby causing an inhibition of trophoblasts invasion	[58]
Acute promyelocytic leukemia	Decreased PAI-1 and plasminogen, and increased tPA, uPA, and uPAR leads to hyperfibrinolysis	[59]
Inflammation	Plasminogen, plasminogen activators, and inhibitors have been identified in exudates and extracts of inflamed tissue; endotoxin-stimulated macrophages hydrolyze fibrin by a plasmin-mediated process in the absence of plasminogen activator	[60,61,62]

**Table 2:** Overview of the existing plasmin generation assays and their major properties and applications.

	NHA	STA	STPGA	PGA
Substrates (thrombin and plasmin)	Bz-AGR-AMC and bis-(CBZ-FR)-Rho	Boc-VPR-AMC and Boc-EKK-AMC	Z-GGR-AMC and Boc-EKK-AMC	Boc-EKK-AMC
Specificity of the plasmin substrate	Plasmin, thrombin, factor Xa	Plasmin	Plasmin	Plasmin
Calibration method	Calibration with plasmin	-	Michaelis–Menten equation using the obtained values of AMC production rate	Calibration with α_2_M-plasmin
Method to obtain plasmin curve	Comparing the arbitrary fluorescence values to a calibration curve	Subtracting the fluorescence reading of the blank well	Plasmin is calculated using the obtained values of AMC production rate	Comparing the arbitrary fluorescence values to a calibration curve
Correction for substrate consumption/inner filter effect	+/+	−/−	−/+	+/+
tPA (μg/mL)	0.38	0.45	0.7	0.31 or 1.25
TF (pM)	0.3	5	4.5	0.5 or 1
Plasma volume	80	90	80	30
Published applications in health and diseases	-PG in patients with plasminogen and PAI-1 deficiency [92] -PG in hemophilia A patients [93] -PG in patients with rare bleeding disorders [81] -PG in patients with angioedema [82]	-Abnormalities in fibrinolysis in adults and children [83] -PG in human plasma with fibrinolytic deficiency [84]	-Inhibitory effect of C-1 inhibitor [85]	-PG in mice model of diet-induced obesity [44] -PG in mice with fibrin(ogen) deficiency [44] -Pharmacodynamics of TXA [88] -PG in coronavirus disease-2019 patients [94,95]

NHA (Nijmegen hemostasis assay), STA (simultaneous thrombin and plasmin generation assay), STPGA (simultaneous thrombin plasmin generation assay), PGA (plasmin generation assay). Boc-EKK-AMC (Boc-Glu-Lys-Lys-AMC), bis-(CBZ-FR)-Rho (bis-(CBZ-L-phenylalanyl-L-arginine amide)-rhodamine); α_2_M-plasmin (α_2_-Macroglobulin-plasmin).

## Data Availability

Not applicable.
